# Bromide of Potassium

**Published:** 1869-05

**Authors:** 


Bromide of Potassium.—In the *Union Médicale* of January 21st, it is stated that the last number of the Reports of the Medico-Chirurgical Society of Bordeaux contains an account of a case of epilepsy, in which the patient, a woman, took the bromide of potassium in amounts varying from thirty grains to one ounce per diem, for a period of about one year. At the end of this time she died, as is alleged, from the debilitating effects of the salt.

